# A Recombinant Human Adenovirus Type 5 (H101) Combined With Chemotherapy for Advanced Gastric Carcinoma: A Retrospective Cohort Study

**DOI:** 10.3389/fonc.2021.752504

**Published:** 2021-12-09

**Authors:** Ran Zhang, Yanxin Cui, Xin Guan, Xiangjun Jiang

**Affiliations:** Department of Gastroenterology, Qingdao Municipal Hospital, Qingdao University, Qingdao, China

**Keywords:** H101, chemotherapy, advanced gastric carcinoma, survival, response rate

## Abstract

**Background:**

This retrospective cohort study aimed to evaluate the clinical outcomes of H101 combined with chemotherapy for advanced gastric carcinoma (GC) patients.

**Methods:**

The advanced GC patients, who were treated with H101 and/or chemotherapy, were enrolled and divided into three groups according to treatment method. The clinical characteristics of patients, clinical short-term and long-term outcomes, followed up, and complication were analyzed.

**Results:**

A total of 95 patients (30 patients in group A were treated with H101, 33 in group B patients were treated with chemotherapy, 32 patients in group C were treated with H101 combined with chemotherapy) were retrospectively reviewed. The disease control rate (DCR) and overall response rate (ORR) were significantly greater in group C (81.3% and 50.0%) than in groups A (63.3% and 30.0%) and B (66.7% and 33.3%, all *p* < 0.05). The 1- and 2-year survival rates and progression-free survival were significantly greater in group C than in groups A and B (all *p* < 0.05). There was no significant difference in complication among the three groups. At dose levels of 0.5 × 10^12^ vp/day, 1.0 × 10^12^ vp/day, and 1.5 × 10^12^ vp/day, complications were not increased as increased of dose.

**Conclusions:**

H101 combined with chemotherapy may be a potential therapeutic option for patients with advanced GC, and prospective studies with proper assessment of toxicity will be needed in the future.

## Introduction

Gastric carcinoma (GC) is one of the most common malignant tumors in digestive system. In 2020, the new and death cases of GC were approximately 1,090,000 and 770,000 worldwide, respectively, making it the fifth most commonly diagnosed cancer and the third most common cause of cancer death globally (1). Especially, in China, according to the Global Cancer Observatory in 2020, 478,000 new cases and 374,000 death cases of GC occurred in China, accounting for 44% and 49% of the new and death cases worldwide, respectively (2). The high mortality is mainly due to most patients were with late-stage GC when diagnosed (3). Presently, the treatment modalities of GC mainly include surgery, chemotherapy, targeted therapy, and immunotherapy. The early-stage GC patients is recommended surgery as a curative approach, while advanced GC are mainly treated by chemotherapy, but with a poor prognosis, only 25%–30% 5-year overall survival (OS) rate (4, 5). Many available targeted drugs are limited in efficacy and cannot maintain for long time, due to complicated tumor microenvironment and instability of genes (6). Immunotherapy needs to select appropriate population according to specific molecular markers, and some GC patients have good clinical efficacy after initial immunotherapy, but may have recurrence (7). These drawbacks indicate the new strategies for advanced GC are urgently needed. Oncolytic virus (OV) therapy is poised to be one of the leading treatments for cancer, due to OVs offering the attractive therapeutic combination of tumor-specific cell lysis together with immune stimulation to kill cancer cells, leaving nonmalignant cells unharmed (8).

**Figure 1:**
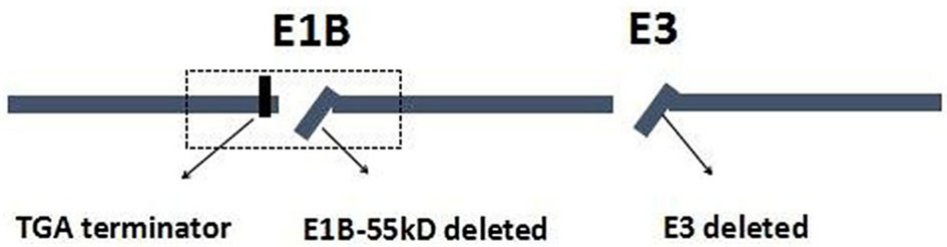
Schematic diagram of constructed adenovirus (H101). E, early region of adenovirus genome.

Wild-type *p53* gene, as a tumor suppressor gene, plays an important role in maintaining normal growth and inhibiting malignant cell proliferation. For *p53* gene mutations, they could lead to an inability to promote apoptosis and the loss of inhibiting cell proliferation and cause excessive cell proliferation and blocks DNA damage repair (9, 10). Previous studies have reported that *p53* mutations were more frequently found in GC, especially in advanced GC or metastasis GC (11). The *p*53 mutations have been related to the worse prognosis and resistance to standard chemotherapeutics in most tumor types, including GC (11, 12). The *p*53 has been extensively studied because of its inhibitory effect on tumorigenesis and is considered to be a promising treatment for cancers. ONYX-015, for example, as an E1B gene-defective adenovirus, was the first genetically engineered OV to be tested in humans; it would selectively replicate and destroy tumor cells carrying mutations of the *p*53 tumor suppressor gene. Furthermore, some studies revealed that ONYX-015 is remarkably safe and effective in the treatment of head and neck cancer, and that the antitumor efficacy could be further enhanced in combination with chemotherapy (13–15). H101, as a recombinant human adenovirus type 5, is similar to ONYX-015, in which the gene encoding the 55-kDa E1B protein responsible for *p53* binding and inactivation has been deleted (16). The H101 also contains a deletion of a 78.3–85.8-μm gene segment in the E3 region. The E3 region is related to the inhibition of host immunity, which enhances the virus replication and spread in the tumor (**Figure 1**) (17, 18).

In 1998, H101, produced by Shanghai Sunway Biotech, initiated the preclinical study in China (19). In a phase II clinical trial, H101 resulted in tumor regression of advanced cancers, with the activated host immune system and enhanced cell-medicated immune responses, indicating that H101 may exert its antitumor effect by promoting the host immune system, especially the cell-medicated immune responses (20). Previously, Wang et al. (18) have summarized the detailed clinical trial of H101 in head and neck cancer, providing information of other ongoing OV clinical trials in China. Recently, some clinical studies have shown that H101 is effective in the treatment of esophageal carcinoma, nasopharyngeal carcinoma, lung carcinoma, and liver carcinoma (21–25). Moreover, the combination of H101 with chemotherapy is superior to chemotherapy alone in delaying the progression of advanced malignant tumors and extending the survival of patients with advanced carcinoma (26, 27). Although the anticancer activity of H101 has been confirmed by extensive intratumoral injection, its clinical efficacy on GC is rarely reported. Therefore, this retrospective cohort study was conducted to evaluate the clinical outcomes of H101 intratumor injection with or without combination with chemotherapy in the treatment of advanced GC.

## Materials and Methods

### Patients

The study was approved by the Ethics Committee of Qingdao Municipal Hospital and performed in accordance with the Helsinki Declaration of 1975 (No. 2018-030). Written informed consent was obtained from all patients before treatment. The patients who were diagnosed with advanced GC and treated with H101 and/or chemotherapy as an initial therapy at the Department of Gastroenterology of Qingdao University Affiliated Qingdao Municipal Hospital (Qingdao, China) between September 5, 2012 and May 20, 2018 were retrospectively studied. Diagnosis of GC was based on the histological assessment of biopsies taken during upper endoscopy. Inclusion criteria include the following: (1) patients were 18–80 years old; (2) patients were unable or refused to undergo surgical treatment; and (3) the lesion site was suitable for intratumoral injection. The patients were excluded if they met the following criteria: (1) had uncontrolled active infection, coagulation abnormality, or serious liver, kidney, or other organ dysfunction; (2) failed to complete the treatment; (3) received surgery, targeted therapy, immunotherapy, or other therapy after H101 and/or chemotherapy. Patients were divided into three groups according to treatment methods: group A, H101 alone; group B, chemotherapy alone; and group C, H101 combined with chemotherapy.

### Treatment Procedures

The treatments were performed according to the uniform protocols recommended by the National Comprehensive Cancer Network (NCCN) practice guidelines for gastric cancer (28). Before endoscopy, all patients underwent laboratory tests and electrocardiogram with fasting, administration of proton pump inhibitors, and nutrition supplementation. The tumor size and the number of lesions were evaluated by endoscopy. H101 (−20°C, Shanghai Sunway Biotech, Shanghai, China) was then dissolved with normal saline to 30% of the estimated tumor volume at room temperature, was peritumorally injected via endoscopy according to the manufacturer’s instructions, and these injections were repeated 21 days as one treatment cycle. The specifications of H101, including the titer, sterility, and general safety, were tested by the National Institute for the Control of Pharmaceutical and Biological Products (Beijing, China) that followed the standard biosecurity and institutional safety procedures (29).

The doses of H101 depended on tumor size and the number of lesions: (1) 0.5 × 1012 virus particles (vp)/day (1 unit) for patients with one lesion with a maximum diameter of ≤5 cm; (2) 1.0 × 1012 vp/day (2 units) for patients with one lesion with a maximum diameter of 5-10 cm or two lesions with a sum of the diameters of 5-10 cm; (3) 1.5 × 1012 vp/day (3 units) for patients with one lesion with a maximum diameter >10 cm or ≥ three lesions; (4) for patients with two or more lesions, the dose of H101 for each lesion was further decided by the proportion and size of the different lesions. The number of cycles of H101 was determined according to the instructions for the use of H101 and patients’ effect after injection. After injection of H101, renin (0.1 mg/ml) and thrombin (10-100 unit/mL) were sprayed to stop the bleeding.

For chemotherapy method, oxaliplatin, 130 mg/m^2^, was administered by intravenous drip on day 1 and Tegafur (Tegafur, Qilu Pharmaceutical (Hainan) Co., Ltd, Haikou, China), 80 mg/m^2^, was taken orally after meals twice a day on days 1–14 in a row with 21 days for a cycle for three cycles. For H101 combined with chemotherapy method, the above mentioned procedures were followed.

### Clinical Evaluation and Follow-Up

Short-term outcome was assessed based on the new Response Evaluation Criteria in Solid Tumors (RECIST) (30), which include complete response (CR, disappearance of all target lesions), a partial response (PR, the sum of all of the length-to-diameter ratio of the target lesion was reduced by 30% or more), stable disease (SD, all of the target lesions changed between PR and PD), and progressive disease (PD, the sum of all of the length to diameter ratio of the target lesion increased by at least 20%, and the absolute value of total length to diameter increased more than 5 mm, or new lesions appear). The disease control rate (DCR) was the proportion of the total number of CR+PR+SD patients treated to the total number of cases. The overall response rate (ORR) was the proportion of the total number of CR+PR patients treated compared to the total number of cases. After treatment, CT scanning and gastrointestinal endoscopy were performed for all patients, and the RECIST was to evaluate the tumor response. Long-term outcome was analyzed by calculating 1- and 2-year overall survival (OS) and progression-free survival (PFS), the median OS and median PFS, the upper quartile (Q_3_) and lower quartile (Q_1_) of OS and PFS, and the interquartile range (IQR = Q_3_–Q_1_) of OS and PFS. The complications, which were defined as any manifestations that occurred during the period of treatment or follow-up, were recorded. All patients were followed up at least every 2 months in the first year after treatment and every 3 months until death or loss of follow-up. The latest follow-up date for this study was September 28, 2018.

### Statistical Analysis

Data were expressed as mean ± standard deviation (SD) or median (range), where appropriate. SPSS version 21 software (SPSS Inc., Chicago, IL, USA) was used to analyze the data. Analysis of variance was used to compare the difference in numerical variables with least significant difference as the *post-hoc* test. The Chi-square test was used to compare the differences between the categorical variables in the groups. Moreover, Kaplan-Meier analysis was used to compare the survival between the groups. A *p*-value <0.05 was considered statistically significant.

## Results

### Patients’ Characteristics

**Figure 2:**
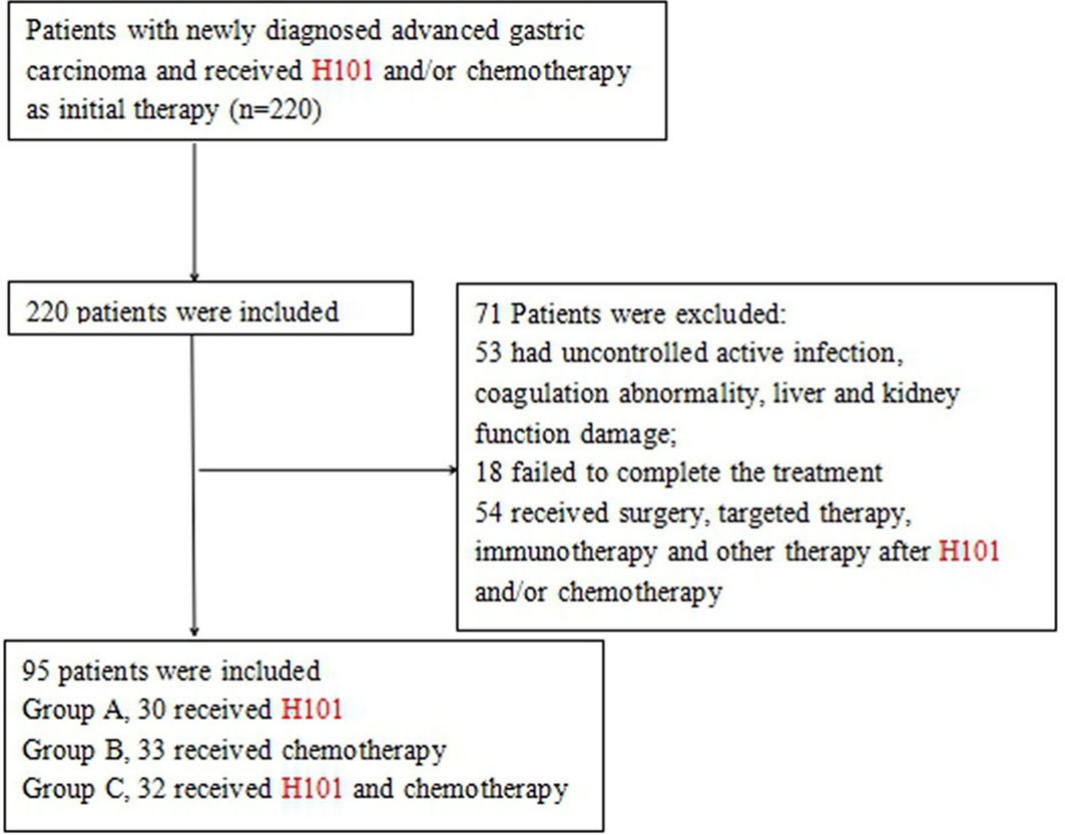
Flowchart of the patient selection process.

Between September 5, 2012 and May 20, 2018, a total of 220 patients were diagnosed with advanced GC and received H101 and/or chemotherapy. Among these patients, 53 patients had uncontrolled active infection, coagulation abnormality, and liver and kidney function damage; 18 patients failed to complete the treatment; and 54 patients received surgery, targeted therapy, immunotherapy, and other therapy after H101 and/or chemotherapy. Finally, 95 patients, 56 males and 39 females aged 45–80 years (67.2 ± 9.8), were included (**Figure 2**). The main clinical symptoms of patients were difficulty eating, upper gastrointestinal bleeding, weight loss, fatigue, appetite loss, nausea and vomiting, and abdominal pain. All patients were at stage III/IV GC and not suitable for surgical resection: 30 cases were treated with H101, including 17 males and 13 females, aged from 47 to 78 years (group A); 33 cases received chemotherapy, including 19 males and 14 females, aged 45–76 years (group B); and 32 cases were treated with H101 combined with chemotherapy, including 20 males and 12 females, aged 48 to 80 years (group C). The main characteristics of the study population are shown in **Table 1**. There was no significant difference among the three groups in terms of age, gender, primary tumor location, pathological type, depth of invasion hepatic metastasis, and GC stage.

**Table 1:** Clinical characteristics of patients treated with recombinant human adenovirus type 5, chemotherapy and H101 combined with chemotherapy (*n* = 95).

Clinical characteristics	Total (*n* = 95)	Group A (*n* = 30)	Group B (*n* = 33)	Group C (*n* = 32)	*p*-value
Age (year)	67.2 ± 9.8	66.7 ± 7.4	65.0 ± 8.8	69.9 ± 6.8	0.846
Gender (*n*)					0.879
Male	56 (58.9%)	17 (56.7%)	19 (57.6%)	20 (62.5%)	
Female	39 (41.1%)	13 (43.3%)	14 (42.4%)	12 (37.5%)	
Primary tumor location (*n*)					0.701
Fundus	18 (18.9%)	6 (20.0%)	7 (21.2%)	5 (15.6%)	
Body	28 (29.5%)	9 (30.0%)	9 (27.3%)	10 (31.3%)	
Pylorus	49 (51.6%)	15 (50.0%)	17 (51.5%)	17 (53.1%)	
Pathological type (*n*)					0.837
Adenocarcinoma	85 (89.5%)	26 (86.7%)	30 (90.9%)	29 (90.6%)	
Signet-ring cell carcinoma	10 (10.5%)	4 (13.3%)	3 (9.1%)	3 (9.4%)	
Depth of invasion (*n*)					0.619
T3	27 (28.4%)	7 (23.3%)	9 (27.3%)	11 (34.4%	
T4	68 (71.6%)	23 (76.7%)	24 (72.7%)	21 (65.6%	
Lymph node metastasis (*n*)					0.947
N1	24 (25.3%)	8 (26.7%)	7 (21.2%)	9 (28.1%)	
N2	41 (43.2%)	13 (43.3%)	14 (42.4%)	14 (43.8%)	
N3	30 (31.5%)	9 (30.0%)	12 (36.4%)	9 (28.1%)	
Hepatic metastasis (*n*)					0.935
Yes	50 (52.6%)	15 (50.0%)	18 (54.5%)	17 (53.1%)	
No	45 (47.4%)	15 (50.0%)	15 (45.5%)	15 (46.9%)	
Stage of gastric carcinoma (*n*)					0.710
III	35 (36.8%)	10 (33.3%)	14 (42.4%)	11 (34.4%)	
IV	60 (63.2%)	20 (66.7%)	19 (57.6%)	21 (65.6%)	

Group A, H101 alone; group B, chemotherapy alone; group C, H101 combined with chemotherapy; N1, 1–2 lymph node metastasis; N2, 3-6 lymph node metastasis; N3, ≥7 lymph node metastasis.

### Clinical Outcomes and Follow-Up

**Figure 3:**
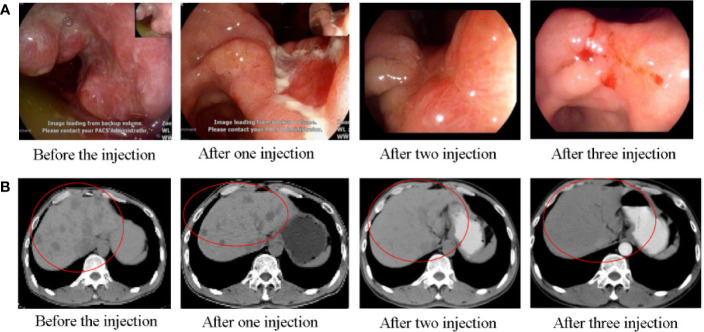
A 51-year-old male patient was diagnosed with T2N1M1 stage gastric carcinoma (GC), involving the entire gastric angle and anterior wall of lesser curvature of gastric antrum. **(A)** The gastroscopy showed that there were huge ulcer-like tumors in the whole gastric angle and the anterior wall of the lesser curvature of the gastric antrum. The bottom of the tumor was uneven, nodular, and covered with white moss, and the edge was raised, with erosion, hyperemia, edema, poor peristalsis, and hard texture. After treatment, the huge ulcer-like tumor was reduced and healed, and the mucosa was gathered, the edge was raised, congestion and edema were reduced, and the tumor volume was significantly reduced. **(B)** Contrast-enhanced CT of the upper abdomen, before endoscopic injection treatment, revealed multiple low-density foci in the liver parenchyma before endoscopic injection, with unclear boundaries, the largest of which was about 2.5 cm in diameter. After the treatment, CT examination showed that the multiple metastases in the liver were significantly reduced and smaller than before and some of the low-density lesions in the liver disappeared.

Overall, 95 patients were evaluable. In group A (*n* = 30 cases) with effective H101 injection, there were one CR and 11 PDs for the control lesions, respectively. Whereas, the combination of H101 injection with chemotherapy in group C (*n* = 32 cases, four CRs and six PDs) was more effective than H101 injection alone in group A (all p < 0.05, **Table 2**). The DCR and ORR were 63.3%, 66.7%, and 81.3% and 30.0%, 33.3%, and 50.0%, respectively, in groups A, B, and C, with the rates being significantly greater in group C than in groups A and B (both *p* < 0.05, **Table 2**). After treatment, most patients had tumors involving pylorus lead to symptoms of obstruction, which was significantly improved in three groups, meanwhile H101 or/and chemotherapy had antitumor effect on metastatic lesions. **Figure 3** shows the regression/response after the injection of H101 in one typical case.

**Table 2:** Short-term outcomes of H101, chemotherapy, and H101 combined with chemotherapy for advanced gastric carcinoma.

	Group A (*n* = 30)	Group B (*n* = 33)	Group C (*n* = 32)	*p*-value
Response assessment after treatment				
Complete response	1 (3.3%)	2 (6.1%)	4 (12.5%)^*^	0.022
Partial response	8 (26.7%)	9 (27.3%)	12 (37.5%)	0.168
Stable disease	10 (33.3%)	11 (33.3%)	10 (31.3%)	0.941
Progressive diseases	11 (36.7%)	11 (33.3%)	6 (18.7%)^*^	0.014
Disease control rate	19 (63.3%)	22 (66.7%)	26 (81.3%)^*^	0.014
Overall response rate	9 (30.0%)	11 (33.3%)	16 (50.0%)^*^	0.007

Group A, H101; Group B, chemotherapy; Group C, H101 combined with chemotherapy. ^*^p < 0.05 compared with group A.

**Figure 4:**
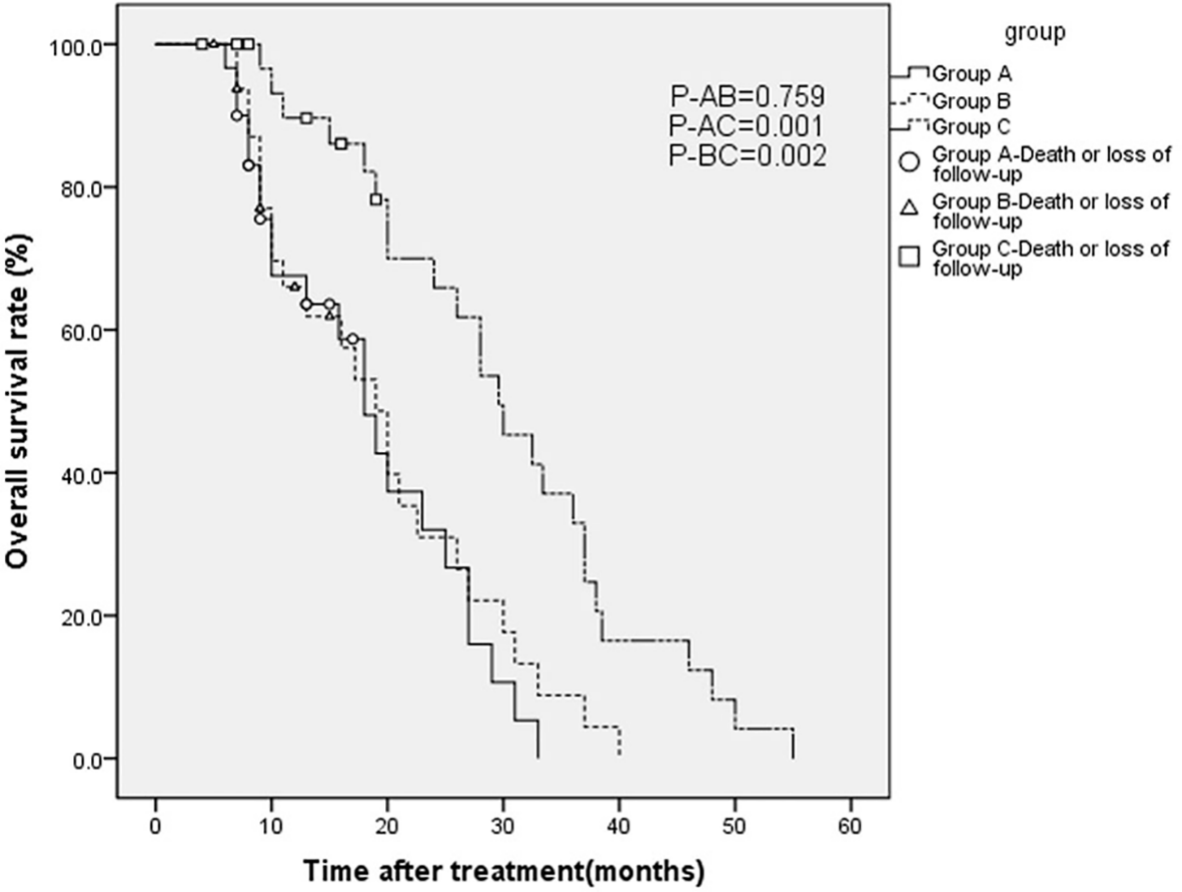
Overall survival (OS) curves estimated by the Kaplan-Meier method for patients with advanced gastric carcinoma who were treated with H101 and/or chemotherapy.

**Figure 5:**
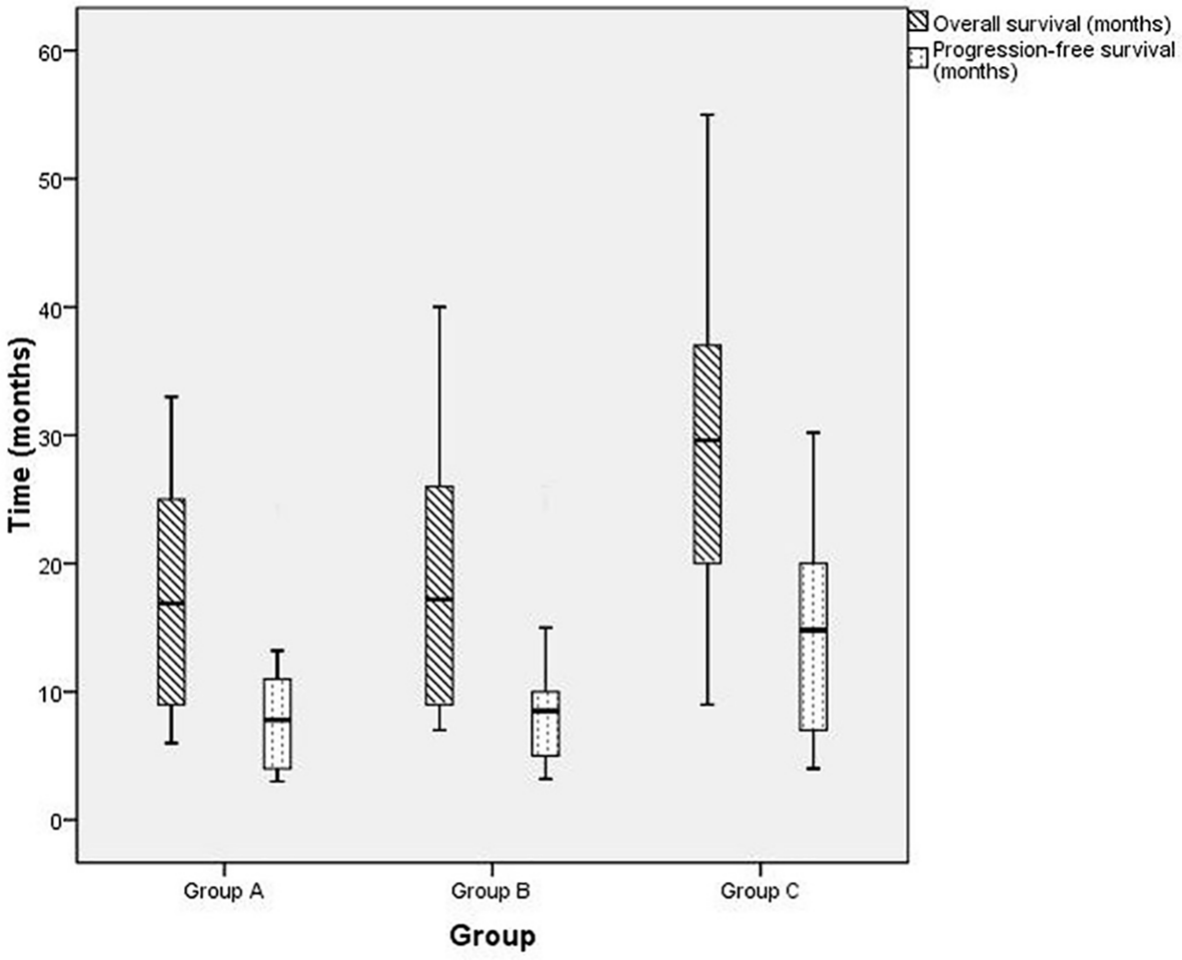
The box plot of overall survival (OS) and progression-free survival (PFS) for patients with advanced gastric carcinoma who were treated with H101 and/or chemotherapy. Group A (OS: Q_3_ = 25.5, Q_1_ = 8.75, median OS = 16.9, PFS: Q_3_ = 11.375, Q_1_ = 4.0, median PFS = 7.8, IQR = 7.375), group B (OS: Q_3_ = 26.5, Q_1_ = 9.0, median OS = 17.2, PFS: Q_3_ = 11.15, Q_1_ = 5.0, median PFS = 8.5, IQR=6.15), group C (OS: Q_3_ = 37.5, Q_1_ = 19.5^*^, median OS = 29.6^*^, PFS: Q_3_ = 20.5, Q_1_ = 7.0, median PFS = 14.8^*^, IQR = 13.5^*^). ^*^ *p* < 0.05 compared with group A or B.

As of September 28, 2018, five, four, and four patients were known to be dead and three, four, and three patients were lost to follow-up in groups A, B, and C, respectively. Thus, 22, 25, and 25 cases in groups A, B, and C, respectively, were followed up for 2 years and longer. Generally, patients in group C survived longer than those in groups A and B, as analyzed by the Kaplan-Meier method (**Figure 4**). Furthermore, as **Table 3** shows, in 1- and 2-year OS and PFS, the median OS and median PFS in group C were significantly greater than those in the other two groups (all *p* < 0.05). In addition, Q_3_ and Q_1_ of OS and PFS of group C were significantly higher (OS: Q_3_ = 37.5, Q_1_ = 19.5, PFS: Q_3_ = 20.5, Q_1_ = 7.0) than that of group A (OS: Q_3_ = 25.5, Q_1_ = 8.75, PFS: Q_3_ = 11.375, Q_1_ = 4.0) and group B (OS: Q_3_ = 26.5, Q_1_ = 9.0, PFS: Q_3_ = 11.15, Q_1_ = 5.0) (all *p* < 0.05). They were not significantly different between groups A and B. IQR of PFS was significantly higher in group C (13.5) than in group A (7.375) and group B (6.15); IQR of OS was not significantly different in three groups (**Figure 5**).

**Table 3:** Long-term outcomes of H101, chemotherapy, and H101 combined with chemotherapy for advanced gastric carcinoma.

	Group A (*n* = 30)	Group B (*n* = 33)	Group C (*n* = 32)	*p*-value
Overall survival				
Median (months)	16.9 (13.0–20.9)	17.2 (12.5–19.4)	29.6 (22.1–31.2)^*^	
1-year (%)	59.1	60.0	88.0^*#^	0.000
2-year (%)	27.3	28.0	60.0^*#^	0.000
Progression-free survival				
Median (months)	7.8 (6.1–10.5)	8.5 (6.9–11.7)	14.8 (9.4–15.3)^*^	
1-year (%)	22.7	24.0	52.0^*#^	0.000
2-year (%)	4.5	8.0	20.0^*#^	0.001

Group A, H101; Group B, chemotherapy; Group C, H101 combined with chemotherapy. ^*^p < 0.05 compared with group A; ^#^p < 0.05 compared with group B.

### Complications

No serious complication or severe toxicity was reported during the treatment or follow-up period. The complication rates of the three groups are summarized in **Table 4**. Fever was more frequently observed in groups A and C than in group B, whereas nausea and vomiting, constipation, granulocytopenia, anemia, and hair loss occurred more commonly in groups B and C than in group A but without significant differences between groups B and C. All complications were alleviated by symptomatic treatment. Furthermore, the incidence of complications did not vary with the dosage changes (0.5 × 10^12^ vp/day, 1.0 × 10^12^ vp/day, and 1.5 × 10^12^ vp/day), correspondingly (**Table 5**).

**Table 4:** Complications of H101, chemotherapy, and H101 combined with chemotherapy for advanced gastric carcinoma.

Complications	Group A (*n* = 30)	Group B (*n* = 33)	Group C (*n* = 32)	*p*-value
Nausea and vomiting	8 (26.7%)	25 (75.8%)^*^	24 (75.0%)^*^	0.000
Diarrhea	13 (43.3%)	14 (42.4%)	13 (40.7%)	0.960
Constipation	4 (13.3%)	11 (33.3%)^*^	12 (37.5%)^*^	0.000
Granulocytopenia	1 (3.3%)	18 (54.5%)^*^	19 (59.4%)^*^	0.000
Anemia	2 (6.6%)	15 (45.5%)^*^	16 (50.0%)^*^	0.000
Hair loss	0 (0.0%)	17 (51.5%)^*^	17 (53.1%)^*^	0.000
Fever	25 (83.3%)	14 (42.4%)^*^	27 (84.4%)^#^	0.000
Overall	25(83.3%)	31 (93.9%)^*^	29 (90.6%)	0.034
Alleviated	25 (100%)	31 (100%)	29 (100%)	–

Group A, H101; Group B, chemotherapy; Group C, H101 combined with chemotherapy. ^*^p < 0.05 compared with group A. ^#^p < 0.05 compared with group B.

**Table 5:** Complications of the three dose levels of recombinant human adenovirus type 5 (H101) in groups A and C.

Complications	Group A at various doses (vp/day) (*n* = 30)				Group C at various doses (vp/day) (*n* = 32)			
	0.5 × 10^12^ (*n* = 5)	1.0 × 10^12^ (*n* = 13)	1.5 × 10^12^ (*n* = 12)	*p*-value	0.5 × 10^12^ (*n* = 3)	1.0 × 10^12^ (*n* = 15)	1.5 × 10^12^ (*n* = 14)	*p*-value
Nausea and vomiting	1 (20.0)	4 (30.1)	3 (25.0)	0.503	2 (66.7)	12 (80.0)	10 (71.4)	0.107
Diarrhea	2 (40.0)	6 (46.2)	5 (41.7)	0.683	1 (33.3)	6 (40.0)	6 (42.3)	0.389
Constipation	0 (0.0)	2 (15.4)	2 (16.7)	0.000	1 (33.3)	7 (46.7)	4 (33.3)	0.062
Granulocytopenia	0 (0.0)	1 (7.7)	0 (0.0)	0.000	2 (66.7)	8 (53.3)	9 (64.2)	0.101
Anemia	0 (0.0)	1 (7.7)	1 (8.3)	0.015	1 (33.3)	8 (53.3)	7 (50.0)	0.009
Hair loss	0 (0.0)	0 (0.0)	0 (0.0)	–	1 (33.3)	8 (53.3)	8 (57.1)	0.001
Fever	4 (80.0)	11 (84.6)	10 (83.3)	0.643	3 (100.0)	12 (80.0)	12 (85.7)	0.000
Overall	4 (80.0)	11 (84.6)	11 (91.7)	0.052	3 (100.0)	13 (86.7)	13 (92.9)	0.048

Data were expressed as number (percentage). Group A, H101; Group C, H101 combined with chemotherapy.

## Discussion

In this retrospective cohort study, the clinical outcomes of three treatment modalities of advanced GC were compared. Regarding short-term outcomes, the rates of CR and PR were significantly higher in patients treated with H101 combined with chemotherapy than in those treated with chemotherapy alone or H101 alone, whereas there was no significant difference in the rate of SD among the three groups. Correspondingly, the rate of PD was less in patients treated with H101 combined with chemotherapy than in the other two groups. Therefore, the DCR and ORR were both higher in patients treated with H101 combined with chemotherapy than in those treated with H101 alone or chemotherapy alone. These findings indicated that H101 combined with chemotherapy was beneficial in terms of short-term clinical outcome in the treatment of advanced GC. However, H101 alone had no obvious advantage regarding short-term outcomes compared with chemotherapy alone. In this study, the DCR of patients who were treated with H101 combined with chemotherapy was higher than that of a previous study reported (81.3% vs. 73.44%) by Lu et al. (20), which might indicate when combined with chemotherapy, the short-term outcomes of H101 through upper endoscopy appears to be better than intravenous drips combined with chemotherapy.

Oxaliplatin is a diaminocyclohexane-containing third-generation platinum compound; it was first patented in 1976 and approved for medical use in 1996 (31). Oxaliplatin has clinical activity as a monosubstance, but it is usually used in combination with other chemotherapeutic drugs to form some of the most common chemotherapeutic schemes in modern oncology (32). Tegafur, which is widely used in Asia, has a similar effect with other chemotherapy regimens. One previous meta-analysis has summarized the effect of a tegafur-based regimen compared with a surgery-alone control, it is suggested that chemotherapy with a tegafur-based agent after surgery can improve the survival of patients with curatively resected GC (33). The mechanism of action of H101 in killing tumor cells is different from that of chemotherapy, but they have synergistic effect. The clinical study has shown that H101 combined with traditional chemotherapy is effective in the treatment of various solid tumors, and the efficacy is better than that of chemotherapy alone (20). However, the efficacy and safety of endoscopic tumor injection of H101 in the treatment of gastric cancer have not been reported. H101 can change its biological activity after acting on tumor cells, making it easier for chemotherapy drugs to enter tumor cells and increasing the effect of chemotherapy to kill tumors. In this study, for advanced GC patients who were treated with the H101 combined with chemotherapy (oxaliplatin + tegafur), H101 was injected into the tumor locally and uniformly under endoscopic operation. After treatment, it was observed visually under an endoscope that the lesion volume of the patient was smaller than before, the congestion and edema of local tissues were reduced, the patient’s self-conscious obstruction caused by tumor was significantly reduced, and the feeding condition was improved. All the above further proved the H101 combined with chemotherapy was effective to treat GC.

According to a report by Peng et al. (34), the median OS and PFS of advanced GC patients with conventional chemotherapy was 6.2 and 11.5 months. Furthermore, a multiple-center phase II study has reported that the oxaliplatin combining with oral tegafur-uracil (uracil combined with tegafur in a 4:1 ratio) could produce a 50% response rate, PFS of 177 days and OS of 331 days, and showed acceptable activity and manageable toxicity in treating patients with advanced GC (35). In this study, some patients who only received the chemotherapeutic combination of oxaliplatin and tegafur achieved a 33% response rate, 8.5 months PFS and 17.2 months OS. Patients treated with H101 combined with chemotherapy had extended survival compared with those treated with H101 alone or chemotherapy alone; the median OS and PFS with H101 combined with chemotherapy were 29.6 and 14.8 months, respectively, which were nearly twice those with H101 alone or chemotherapy alone. The Q_1_ of OS of patients with H101 combined with chemotherapy were more than twice those with H101 alone or chemotherapy alone, the Q_3_ of OS and PFS and Q_1_ of PFS with H101 combined with chemotherapy were clearly increased than the other groups. Moreover, the IQR of PFS with H101 combined with chemotherapy, H101 alone, and chemotherapy alone were 13.5, 7.38, and 6.15 months, respectively. This might indicate the PFS of H101 alone and chemotherapy alone is more concentrated. These findings indicated that H101 combined with chemotherapy might have substantially better long-term outcomes than H101 alone and chemotherapy alone.

One study has reported that low toxicity was observed in patients with the squamous cell carcinoma of the head and neck after they received intratumoral H101 injection in a dose-escalation manner (from 5.0 × 10^7^ to 1.5 × 10^12^ vp/day) for 5 consecutive days. The most frequent complications were fever, flulike symptoms, and pain at the injection site (19). In our study, fever was also frequently observed in groups A and C, which was higher than that in group B. All complications in groups A and C were not increased following the increasing of H101 dose (0.5 × 10^12^ vp/day, 1.0 × 10^12^ vp/day, and 1.5 × 10^12^ vp/day), which was were similar to previous reports (19, 29). This study further proved H101 combined with chemotherapy can effectively control the growth of gastric malignant tumors and improve the survival rate without increasing complications. The upper endoscopic procedure is relatively simple, and H101 through upper endoscopy combined with chemotherapy has positive clinical value that is worthy of clinical promotion and application. Moreover, the present study also provides a relevant basis for a therapeutic adenovirus combined with chemotherapy in the treatment of GC.

However, this study has some inherent limitations due to being a retrospective study. First, selection bias might be present but cannot be fully assessed for such an observational study. Second, the subjects were all from one hospital, the sample size was relatively small, which weakened the statistical power of the analyses. Third, due to its relatively high cost and patients having doubts about its efficacy and safety, patients’ acceptance of H101 is still relatively low and the clinical application of H101 is not very common. Therefore, a prospective randomized controlled trial with larger samples is urgently needed to obtain more robust clinical data and more convincing results to guide clinical treatment.

In conclusion, H101 combined with chemotherapy may be a potential therapeutic option for patients with advanced GC, and prospective studies with proper assessment of toxicity will be needed in the future.

## Data Availability Statement

The original contributions presented in the study are included in the article/supplementary material. Further inquiries can be directed to the corresponding author.

## Ethics Statement

The studies involving human participants were reviewed and approved by Qingdao Municipal Hospital, Qingdao Affiliated to Qingdao University. The patients/participants provided their written informed consent to participate in this study.

## Author Contributions

Conception and design: RZ. Administrative support: XJ. Provision of study materials or patients: XJ. Collection and assembly of data: RZ, YC and XG. Data analysis and interpretation: RZ and YC. Manuscript writing: all authors. All authors contributed to the article and approved the submitted version.

## Conflict of Interest

The authors declare that the research was conducted in the absence of any commercial or financial relationships that could be construed as a potential conflict of interest.

## Publisher’s Note

All claims expressed in this article are solely those of the authors and do not necessarily represent those of their affiliated organizations, or those of the publisher, the editors and the reviewers. Any product that may be evaluated in this article, or claim that may be made by its manufacturer, is not guaranteed or endorsed by the publisher.
