# Corrigendum: A Recombinant Human Adenovirus Type 5 (H101) Combined With Chemotherapy for Advanced Gastric Carcinoma: A Retrospective Cohort Study

**DOI:** 10.3389/fonc.2022.841156

**Published:** 2022-02-22

**Authors:** Ran Zhang, Yanxin Cui, Xin Guan, Xiang Jun Jiang

**Affiliations:** Department of Gastroenterology, Qingdao Municipal Hospital, Qingdao University, Qingdao, China

**Keywords:** H101, chemotherapy, advanced gastric carcinoma, survival, response rate

In the original article, there were three errors in the text.

Firstly, the dissolution of H101 was incorrectly stated. A correction has been made to **Materials and Methods** section, *“Treatment Procedures”*, Paragraph 1:

“H101 (-20°C, Shanghai Sunway Biotech, Shanghai, China) was then dissolved with normal saline to 30% of the estimated tumor volume at room temperature, was peritumorally injected *via* endoscopy according to the manufacturer’s instructions, and these injections were repeated 21 days as one treatment cycle”.

Secondly, the dose of H101 for patients with one lesion with a maximum diameter of ≤5 cm was incorrectly stated. A correction has been made to **Materials and Methods** section, *“Treatment Procedures”*, Paragraph 2:

“The doses of H101 depended on tumor size and the number of lesions: (1) 0.5 × 10^12^ virus particles (vp)/day (1 unit) for patients with one lesion with a maximum diameter of ≤5 cm; (2) 1.0 × 10^12^ vp/day (2 units) for patients with one lesion with a maximum diameter of 5-10 cm or two lesions with a sum of the diameters of 5-10 cm; (3) 1.5 × 10^12^ vp/day (3 units) for patients with one lesion with a maximum diameter >10 cm or ≥ three lesions; (4) for patients with two or more lesions, the dose of H101 for each lesion was further decided by the proportion and size of the different lesions. The number of cycles of H101 was determined according to the instructions for the use of H101 and patients’ effect after injection. After injection of H101, renin (0.1 mg/ml) and thrombin (10-100 unit/mL) were sprayed to stop the bleeding”.

Thirdly, the statistical comparison of CR and PD between group A and group C was incorrectly stated. A correction has been made to **Results** section, *“Clinical Outcomes and Follow-Up”*, Paragraph 1:

“Whereas, the combination of H101 injection with chemotherapy in group C (*n* = 32 cases, four CRs and six PDs) was more effective than H101 injection alone in group A (all *p <* 0.05, **Table 2**).”

**Table 2:** Short-term outcomes of H101, chemotherapy, and H101 combined with chemotherapy for advanced gastric carcinoma.

	Group A (*n* = 30)	Group B (*n* = 33)	Group C (*n* = 32)	*p*-value
Response assessment after treatment				
Complete response	1 (3.3%)	2 (6.1%)	4 (12.5%)^*^	0.022
Partial response	8 (26.7%)	9 (27.3%)	12 (37.5%)	0.168
Stable disease	10 (33.3%)	11 (33.3%)	10 (31.3%)	0.941
Progressive diseases	11 (36.7%)	11 (33.3%)	6 (18.7%)^*^	0.014
Disease control rate	19 (63.3%)	22 (66.7%)	26 (81.3%)^*^	0.014
Overall response rate	9 (30.0%)	11 (33.3%)	16 (50.0%)^*^	0.007

Group A, H101; Group B, chemotherapy; Group C, H101 combined with chemotherapy. ^*^p < 0.05 compared with group A.

The authors apologize for these errors and state that this does not change the scientific conclusions of the article in any way. The original article has been updated.

## Publisher’s Note

All claims expressed in this article are solely those of the authors and do not necessarily represent those of their affiliated organizations, or those of the publisher, the editors and the reviewers. Any product that may be evaluated in this article, or claim that may be made by its manufacturer, is not guaranteed or endorsed by the publisher.

